# Supplementary figures and images for: The Molecular Evolution of Circadian Clock Genes in Spotted Gar (Lepisosteus oculatus)
Source: Genes (Basel). 2019 Aug 17;10(8):622. doi: 10.3390/genes10080622 (PMC6723592; doi:10.3390/genes10080622)

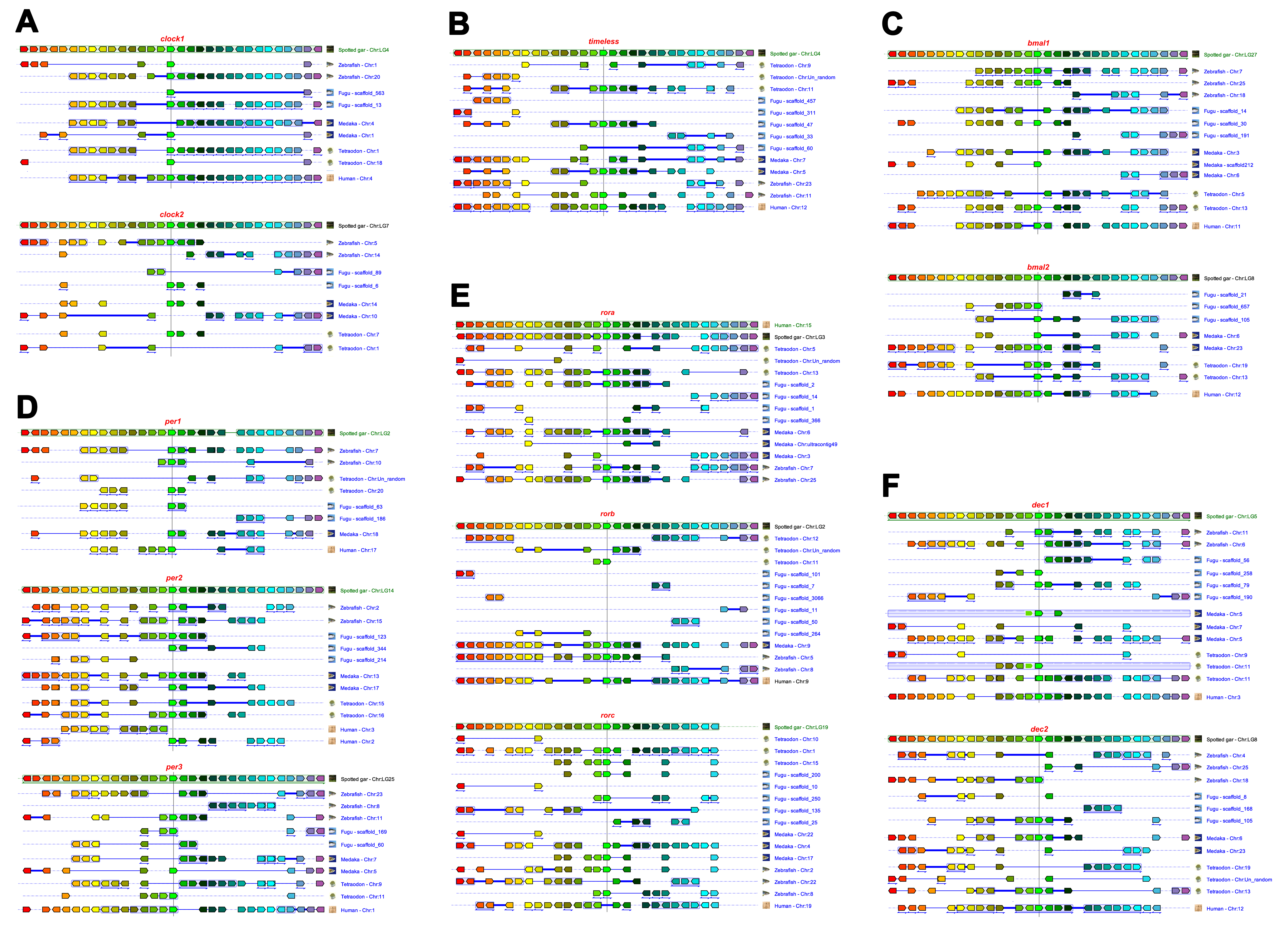

Supplement: Supplementary file 1 [file genes-10-00622-s001.zip › genes-554257-Figure S1.tif]
